# Assessing Parental Competence and Self-Ratings in Management of Pediatric Type 1 Diabetes and Emergency Glucagon Administration—An Exploratory Observational Study

**DOI:** 10.3390/children10081319

**Published:** 2023-07-31

**Authors:** Simone Eisenhofer, Martina P. Neininger, Astrid Bertsche, Wieland Kiess, Thilo Bertsche, Thomas M. Kapellen

**Affiliations:** 1 Drug Safety Center and Clinical Pharmacy, Institute of Pharmacy, Medical Faculty, Leipzig University, Bruederstr. 32, 04103 Leipzig, Germany; simone.eisenhofer@uni-leipzig.de (S.E.); martina.neininger@uni-leipzig.de (M.P.N.); 2 Department of Pediatric Neurology, University Medicine Greifswald, Fleischmannstraße 6, 17489 Greifswald, Germany; astrid.bertsche@med.uni-greifswald.de; 3 Department of Women and Child Health, Hospital for Children and Adolescents and Center for Pediatric Research, University Hospital of Leipzig, Liebigstr. 23, 04103 Leipzig, Germany; wieland.kiess@medizin.uni-leipzig.de (W.K.); thomasmichael.kapellen@medizin.uni-leipzig.de (T.M.K.); 4 MEDIAN Kinderklinik am Nicolausholz Hospital for Children and Adolescents, Elly-Kutscher-Straße 16, 06628 Naumburg (Saale), Germany

**Keywords:** diabetes mellitus, type 1, severe hypoglycemia, hypoglycemia management, glucagon administration, drug handling error, drug safety, error awareness

## Abstract

Background: Parents of pediatric patients with type I diabetes require competence in hypoglycemia management and skills in glucagon administration to deal with potentially life-threatening severe hypoglycemia. We aimed to compare parents’ subjective self-ratings to an objective expert assessment of competences and skills in dealing with severe hypoglycemia. Methods: We interviewed 140 participants to assess their subjective self-ratings. The objective expert assessments used a standardized clinical case scenario of severe hypoglycemia and a practical demonstration of glucagon administration. Results: The participants self-rated their competence in hypoglycemia management as good (5) or very good (6), and their skills in administering glucagon as acceptable (3) [Scale: very poor (1) to very good (6)]. In the standardized clinical case scenario, 1.4% (2/140) of participants named all relevant steps of severe hypoglycemia management. In the practical demonstration of glucagon administration, 92.9% (130/140) of participants committed at least one drug handling error; 52.1% (73/140) committed at least one drug handling error rated with high clinical risk. Conclusions: We found discrepancies regarding participants’ subjective self-ratings compared to their performance in the respective objective expert assessments. These discrepancies indicate a lack of error awareness and the need for intervention studies to improve competence in hypoglycemia management and glucagon administration.

## 1. Introduction

Type 1 diabetes is the most common metabolic disease in pediatric patients [1]. The chronic disease is accompanied by two acute complications: hypoglycemia and ketoacidosis [1]. Hypoglycemia is the most common acute complication of type 1 diabetes and is a potentially life-threatening emergency [1,2,3,4,5]. With a weekly average of two symptomatic hypoglycemic events, hypoglycemia represents a considerable role in the everyday life of patients and their parents [3,4]. Recurring episodes of hypoglycemia can negatively affect patients’ quality of life, health, long-term prognosis, and behavior [2,3,4,5,6,7]. Hypoglycemia is classified into two levels of severity: mild and severe [1,2,5]. Mild hypoglycemia can be treated by oral intake of fast-acting carbohydrates [1,2,5]. In the case of severe hypoglycemia, oral carbohydrate intake is not possible due to somnolence or unconsciousness [1,2,5]. Severe hypoglycemia requires parents to follow a defined plan of action and have adequate competence in hypoglycemia management to resolve the glycemic complication [1,2,5,8]. If the patient is unresponsive or unconscious, parenteral glucagon is part of this plan of action [1,2,3,4,5,8,9,10]. The glucagon-induced rapid increase of blood glucose levels can rescue the patient from the potentially life-threatening situation, and lead to a reduced need for hospitalization and a faster recovery [4,11]. However, for it to work effectively, safe drug administration is crucial. Glucagon is only used occasionally in serious emergency events, which pose a challenging and stressful situation [1,2,3,4,5]. Furthermore, glucagon administration is primarily conducted by medical laypersons [1,2,3,4,10]. During the study period, only glucagon for intramuscular or subcutaneous use (i.m./s.c. glucagon) was available in Germany. It is a complex medicinal product for which medical laypersons need special training due to the multistep reconstitution and parenteral injection [5,9,10]. This is underlined by a study of Yale et al., which showed that the majority of participants were not able to correctly reconstitute i.m./s.c. glucagon and administer the recommended dose [12]. Past studies concerning hypoglycemia and other indications showed that non-medical professionals, such as parents or teachers, felt poorly prepared and reluctant to administer rescue medication [11,13,14,15]. Despite this, a study on epilepsy showed that parents overestimate their skills in administering emergency medication [16]. The mentioned circumstances and the potential deficit of error awareness are barriers to a safe and effective drug administration and could cause serious harm to the patient [2,3,4,5].

Therefore, the aim of this study was to evaluate parents’ subjective self-ratings on their competence in hypoglycemia management and skills in i.m./s.c. glucagon administration. Furthermore, we aimed to objectively assess their competencies and skills by using standardized clinical case scenarios and a demonstration model. Finally, we aimed to analyze potential discrepancies between the parents’ self-assessment and the objective assessment by experts to identify possible approaches for future drug safety optimization strategies.

## 2. Materials and Methods

### 2.1. Participants and Setting

We invited parents of pediatric diabetes patients who had an outpatient appointment at the pediatric diabetes department to take part in this study. Data collection took place from March 2019 to March 2020. Our sample size calculation (*N* = 300, *t* = 1.96, *p* = 0.8, *d* = 0.05) suggested including 135 participants [17]. The pediatric patients had to be under the age of 18 and diagnosed with type 1 diabetes for their parents to be included. Parents had to be legal guardian of the pediatric patients and needed to have sufficient knowledge of the German language. If both parents were present, we interviewed the parent who was most involved in the child’s therapy. Parents whose children had more than one outpatient visit during the study period were only included once. Data collection on parts I–III of the interview (demographic data; subjective self-ratings of competence in hypoglycemia management; subjective self-ratings of skills in glucagon administration) took place at the diabetes department. Data collection on parts IV–VI of the interview (objective expert assessment of competence in severe hypoglycemia management; objective expert assessment of skills in glucagon administration; access to glucagon) took place at the patients’ homes. We preferred conducting data collection on these parts at participants’ homes to gain insight into their everyday life. This was especially important in order to evaluate the storage conditions and expiration date of the glucagon. If participants did not agree to data collection at their home, it took place at the diabetes department, and data on access to glucagon was obtained by phone. The latter was conducted by phone to avoid recall bias and to enable parents to look up the expiration date of the glucagon.

### 2.2. Ethical Considerations

The local ethics committee approved the study protocol. We obtained written informed consent from all participating parents and children over 12 years of age. The approved protocol required pseudonymous data collection. Participants’ personal data are stored under seal at the local university hospital, separated from the data obtained in the interview and the practical demonstration. The identifying patient list will be destroyed after a storage period of 10 years, transforming the pseudonymous data into anonymous data. Contact information (phone number, address) were destroyed directly after use. To ensure privacy and confidentiality, the manuscript contains only anonymized data in aggregate without the use of identifiers. All authors declare to have no conflict of interest. Therefore, neither data collection and analysis nor the draft of the manuscript were in any way interest-driven.

To ensure participants’ safety, we simulated the glucagon administration in a demonstration model. To rule out any potential risk for participants, we used placebo powder, a blunt syringe, and a sponge to imitate administration. We did not use active ingredients or perform physical drug administration.

To facilitate personal benefit for all participants, the study protocol provided individual training concerning the detected hypoglycemia management errors and glucagon drug handling errors. The individual training was not part of the data collection. With this study, we aimed to generate general benefit for pediatric type 1 diabetes patients and their parents by identifying problems in hypoglycemia management and glucagon administration, and to provide knowledge on how to improve respective training.

### 2.3. Interview

We conducted an interview with six parts:

- Part I: Demographic data
- Part II: Subjective self-ratings of competence in hypoglycemia management
- Part III: Subjective self-ratings of skills in glucagon administration
- Part IV: Objective expert assessment of competence in severe hypoglycemia management (standardized clinical case scenario)
- Part V: Objective expert assessment of skills in glucagon administration (practical demonstration using a demonstration model and documentation of drug handling errors)
- Part VI: Access to glucagon

The interview was designed by an expert panel which consisted of clinical pharmacists, diabetologists, and pediatricians. It was based on local hypoglycemia training, national and international guidelines, and the Summary of Product Characteristics and Patient Leaflet of the only available glucagon product available during study period (GlucaGen^®^ HypoKit”, Novo Nordisk Pharma GmbH, Mainz, Germany) [1,2,5,8,9,10]. The interview reflects the consensus of the expert panel and serves as a standardized document for the conduction of the interview and the practical demonstration. To ensure consistent data assessment in the practical demonstration, we used a standardized checklist for the documentation of the demonstration model.

We pretested the interview with clinical professionals (*n* = 5) using cognitive pretests. These clinical professionals were not part of the expert panel and were neither involved in the conception of the study, nor in its conduction. The pretests were followed by a pilot with 11 parents.

To ensure standardization and consistent documentation in the interview and to minimize interviewer effects, all interviews were conducted by one trained clinical pharmacist. This training was based on actual trainings for diabetes patients and their parents by the diabetes specialists from the pediatric diabetes department the study was conducted at. 

#### 2.3.1. Part I: Demographic Data

Part I included demographic data on age, gender, education, and profession of both parents and patients. Unavailable information was obtained from patients’ medical records. We also asked parents about the time of their last training session on hypoglycemia and whether they had already used glucagon in a real-life emergency of their child.

#### 2.3.2. Part II: Subjective Self-Ratings of Competence in Hypoglycemia Management

In part II we asked participants to self-rate their competence on three elements: knowledge about hypoglycemia, ability to identify symptoms of hypoglycemia in their children, and skills in managing hypoglycemia. They rated their competence on the following scale: very poor (1), insufficient (2), acceptable (3), fair (4), good (5), very good (6). In addition, we asked participants about their desire for training on hypoglycemia, using a scale with the items no desire (0), little desire (1), moderate desire (2), great desire (3).

#### 2.3.3. Part III: Subjective Self-Ratings of Skills in Glucagon Administration

In part III, parents were asked to self-rate their skills in glucagon administration on the following scale: very poor (1), insufficient (2), acceptable (3), fair (4), good (5), very good (6). In addition, we asked if they had ever used glucagon before. During the study period, only i.m./s.c. glucagon was available to the enrolled participants. Accordingly, they self-rated their skills regarding i.m./s.c. glucagon.

#### 2.3.4. Part IV: Objective Expert Assessment of Competence in Severe Hypoglycemia Management

Part IV aimed to objectively assess parents’ competence in severe hypoglycemia management. For this reason, we designed a standardized clinical case scenario of severe hypoglycemia and asked parents to indicate the steps they would take to resolve the situation. We expected the following steps: step 1 glucagon administration, step 2 recovery position, step 3 emergency call, and step 4 carbohydrate intake in full consciousness. The order of these steps is not crucial for treatment and was therefore not taken into account. Any further information given by the participants was documented and evaluated by the expert panel. 

#### 2.3.5. Part V: Objective Expert Assessment of Skills in Glucagon Administration

In part V, experts objectively assessed parents’ skills in glucagon administration by using a demonstration model to determine whether glucagon was being administered safely and correctly. Glucagon administration was considered “safe” if it would not cause harm to the patients if administered accordingly in a real-world setting. Glucagon administration was defined as “correct” if the administration would result in complete drug intake by the patient under real-life conditions.

This demonstration model consisted of the practical demonstration of glucagon reconstitution and its administration to a sponge, and a question to the parents about the supposed site of application. During the study period, only i.m./s.c. glucagon was available in Germany. Accordingly, the demonstration model simulated the reconstitution and administration of i.m./s.c. glucagon. The glucagon dummy was provided in original packaging. Parents therefore had access to the package leaflet and short instructions including pictograms. A clinical pharmacist, using a standardized checklist to detect drug handling errors, monitored the practical demonstration. The drug handling errors were defined on the basis of the Summary of Product Characteristics and the Patient Leaflet for the only licensed glucagon product in Germany, “GlucaGen^®^ HypoKit” (Novo Nordisk) [9,10]. An expert panel classified the potential clinical risk of the detected drug handling errors, using a scale with three severities: low (1), moderate (2), and high clinical risk (3). For the final rating of the clinical risk, we calculated the median of the individual ratings. The expert panel consisted of experienced pediatric diabetologists, pediatricians, nutritionists, diabetes specialist nurses, and clinical pharmacists. 

#### 2.3.6. Part VI: Access to Glucagon

If the main data collection took place at participants’ homes, parents were asked to show the clinical pharmacist their stored glucagon. If the main data collection took part at the diabetes department, the clinical pharmacist obtained this information by phone. We documented availability, expiration date, storage duration, and storage temperature. If glucagon was present at the family’s home, stored correctly, and had not expired yet, it was considered as ready for use.

#### 2.3.7. Data Analysis

Frequencies are presented as numbers and percentages. Continuous data are given as median, including first and third quartile (Q25/Q75) and minimum/maximum as appropriate. We used SPSS (Statistical Package for the Social Sciences, Version 26, IBM) for statistical calculations. We used the Mann–Whitney U test to compare the number of drug handling errors committed by parents who used the provided patient information and those who did not. A *p* value ≤ 0.05 was considered to indicate significance.

## 3. Results

### 3.1. Part I: Demographic Data

During the study period, we invited 216 parents to participate in this study, of which 147 gave their informed consent. We were able to perform data collection with 140 parents (Table 1).

### 3.2. Part II: Subjective Self-Ratings of Competence in Hypoglycemia Management

On a scale ranging from 1 (very poor) to 6 (very good), participants’ self-ratings on the three aspects of competence in hypoglycemia management were good to very good: knowledge about hypoglycemia (median; Q25/Q75; min/max: 6; 5/6; 2/6), ability to identify symptoms of hypoglycemia (5; 5/6; 3/6), and skills in managing hypoglycemia (6; 6/6; 4/6). In addition, on a scale ranging from no desire (0) to great desire (3), they showed little desire for training on hypoglycemia (1; 1/2; 0/3).

### 3.3. Part III: Subjective Self-Ratings of Skills in Glucagon Administration

Using a scale ranging from 1 (very poor) to 6 (very good), parents self-rated their skills in glucagon administration as acceptable (median; Q25/Q75; min/max: 3; 2/4; 1/6). In 6/140 (4.3%) cases, the interviewed parent or their partner had previously used glucagon at least once. As only i.m./s.c. glucagon was available in Germany during the study period, the self-ratings relate to i.m./s.c. glucagon.

### 3.4. Part IV: Objective Expert Assessment of Competence in Hypoglycemia Management

**Figure 1:**
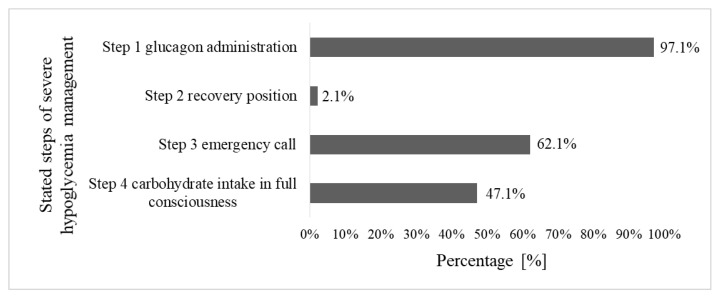
Stated steps of severe hypoglycemia management.

Participants mentioned the four steps of the standardized clinical case scenario used in the objective expert assessment of competence in severe hypoglycemia management as shown in Figure 1. All necessary steps were stated by 1.4% (2/140) of parents.

### 3.5. Part V: Objective Expert Assessment of Skills in Glucagon Administration

Using a demonstration model imitating the administration of i.m./s.c. glucagon and a standardized checklist, we detected 554 drug handling errors (Table 2).

For 3.6% (5/140) of parents, data were not available. In median, the parents conducted 4 (median; Q25/Q75; min/max: 2/6; 0/10) drug handling errors per demonstration [low clinical risk: 1 (0/1; 0/3), moderate clinical risk: 2 (1/3; 0/5), high clinical risk: 1 (0/2; 0/4)]. At least one drug handling error was committed by 92.9% (130/140) of parents and 52.1% (73/140) committed at least one drug handling error rated with high clinical risk. An overdose was administered by 6.4% (9/140) of parents. However, taking into account that an overdose is only possible in patients weighing less than 25 kg (*n* = 19), this results in 47.4% (9/19) overdoses. The provided package leaflet and/or short instructions were used by 43.6% (61/140) of parents. There was no significant difference (*p* = 0.5201; Mann–Whitney U test) in the median of conducted drug handling errors per demonstration between parents who used the package leaflet and/or short instructions and parents who did not.

### 3.6. Part VII: Access to Glucagon

**Figure 2:**
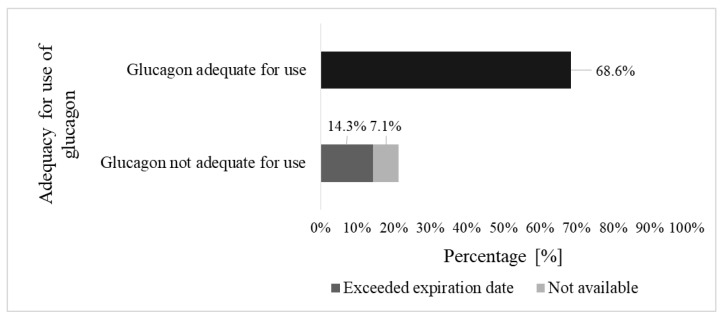
Access to glucagon.

In total, 21.4% (30/140) of parents had no access to glucagon that was adequate for use. Glucagon was determined as adequate for use if the drug was present at their homes, not expired, and stored correctly (Figure 2). For 10.0% (14/140) of the parents, data were not available.

## 4. Discussion

Parents’ subjective self-ratings of their knowledge about hypoglycemia, ability to identify symptoms of hypoglycemia, and skills in managing hypoglycemia were good to very good. In addition, parents subjectively self-rated their skills in glucagon administration as acceptable. In the objective expert assessment of severe hypoglycemia, only very few parents were able to name all relevant steps. In the objective expert assessment of skills in glucagon administration, almost all parents conducted at least one drug handling error. In median, one drug handling error rated as high clinical risk was observed per demonstration. We found discrepancies between participants’ subjective self-ratings and the respective objective expert assessment.

### 4.1. Hypoglycemia Management

Participants’ subjective self-ratings on the three aspects of hypoglycemia management (knowledge about hypoglycemia, ability to identify symptoms of hypoglycemia, and skills in managing hypoglycemia) were good to very good. This indicates a high self-perceived competence in hypoglycemia management.

This opinion was contradicted by our findings in the objective expert assessment of their competence in severe hypoglycemia management. In total, less than 2% of parents named all steps. Therefore, they could not show adequate competence in severe hypoglycemia management. While almost all parents mentioned step 1 glucagon administration, step 2 recovery position was named very rarely. Although generally underutilized, the recovery position is always indicated regardless of the cause of unconsciousness and is specifically recommended by the package leaflet of GlucaGen^®^ HypoKit [10,18,19]. After glucagon administration, the recovery position is even more important as vomiting is a possible adverse drug reaction and can lead to suffocation [9,10]. Despite this, the recovery position is not part of national or international guidelines on hypoglycemia management or local hypoglycemia training [1,2,5,8]. Although taught in local hypoglycemia training, less than two thirds of parents mentioned step 3 emergency call and less than half of parents identified step 4 carbohydrate intake in full consciousness. Both measures are crucial to prevent dangerous second episodes of hypoglycemia [2,3,5,8,18]. To prevent further events of severe hypoglycemia in the long term, parents need to identify the cause of the current episode and develop strategies to avoid it in the future [9,10]. Only 1.4% of participants stated that they did so, showing that parents are not adequately equipped with hypoglycemia prevention strategies.

Our results show that competence in managing severe hypoglycemia needs to be strengthened. In addition, we found a discrepancy between participants’ subjectively self-perceived and objectively expert-assessed competence. This indicates a lack of error awareness, which potentially prevents the affected parents from seeking help from health care professionals.

### 4.2. Safe Glucagon Administration

Less than 5% of parents reported having used i.m./s.c. glucagon before. With an incidence of severe hypoglycemia of 16.6 to 19 per 100 patient years, this percentage seems too low [5]. Additionally, more than 30% of parents did not have access to glucagon that was adequate for use. This is consistent with the literature showing that laypersons are reluctant to administer rescue medications and that they are often unaware of the presence of irregularly used medications [11,13,14,15,16,20]. Parents’ self-assessment that their skills in i.m./s.c glucagon administration are acceptable contrasts with the objective expert assessment; in the demonstration model, almost all parents committed at least one drug handling error and more than half at least one drug handling error rated as high in clinical risk. Remarkably, 15% of parents did not remove the vial’s hard plastic lid before trying to inject the needle. This can lead to a damaged or no longer usable syringe, making a safe drug administration impossible. A further point of interest is that 15% of parents did not inject any solvent into the vial, making it impossible for the glucagon to dissolve. Consequently, they administered pure solvent without any active ingredient. Other parents would put their children in additional danger; almost half of the parents of children weighing less than 25 kg did not conduct the necessary dose reduction. An overdose dramatically increases the risk of an adverse drug reaction, such as vomiting and the resulting choking hazard [9,10]. These results show that in a considerable number of demonstrations, a safe and correct emergency drug administration did not occur. However, for glucagon to achieve its potentially lifesaving effect, it needs to be administered safely and correctly.

There was no difference in the number of drug handling errors between parents who used the package leaflet and/or the short instruction and parents who did not. This indicates that the provided patient information is not suitable to improve administration skills. Furthermore, severe hypoglycemia is a stressful emergency in which it is likely for parents to commit even more drug handling errors than in the study environment. Our data are in line with studies on i.m./s.c. glucagon and on other rescue medications, underling the complexity and error-proneness of emergency drug [4,11,12,13,14,15,16,20].

Intranasal or ready-to-use injectable glucagon and dasiglucagon could solve some of these problems, due to their less complex drug handling [12,21,22,23,24,25,26,27,28,29,30]. Unfortunately, although showing promising results, further data on these alternatives of rescue medication are still limited [29,30] On top of that, their broad use is limited; ready-to-use injectable glucagon and dasiglucagon are currently only approved in the United States of America and the Netherlands [23,24,25]. Intranasal glucagon is approved in the European Union and the United States of America for patients aged 4 years and older [21,22]. By contrast, i.m./s.c. glucagon is approved for patients from birth [9,10]. Until alternatives will be broadly available for all age groups, we need to improve parents’ skills in i.m./s.c. glucagon administration and address their lack of awareness of administration errors. In the future, it is essential to improve glucagon administration skills to ensure drug effectiveness and patient safety in hypoglycemic emergencies.

### 4.3. Clinical Implication: Hypoglycemia Training as a Means to Overcome Lack of Error Awareness

Parents expressed little interest in hypoglycemia training, even though their last training was in median more than two years ago. At the diabetes department, where our study was conducted, hypoglycemia training for parents is offered at the initial manifestation of diabetes and as needed. However, our data suggest that parents lack awareness of errors, which may discourage them from seeking help from health care professionals. It is well known that health training is particularly successful when repeated, e.g., refreshing after three to six months and annually thereafter as suggested in first aid training [1,31]. To implement hypoglycemia and glucagon training more widely, video-guided instructions and virtual training sessions could be used.

To improve training, it should take an individualized and hands-on approach, e.g., with role-playing to simulate severe hypoglycemia, and dummy administration of i.m./s.c. glucagon. In particular, addressing individual errors could sensitize parents to their deficits and thus improve their children’s glycemic control. Such training could be supported by pharmacists and other healthcare professionals who supervise the dummy administration.

### 4.4. New Directions for Future Research

Our results show discrepancies between parents’ self-ratings and objective expert assessments, as found with other emergency medications [16]. As these discrepancies indicate a lack of error awareness, we need future research on how to overcome this barrier to patient safety. Additionally, further studies are needed to improve training on hypoglycemia management and glucagon administration. Intervention studies could help to target specific needs of patients and parents concerning their hypoglycemia and glucagon training. One approach could be the development of a visual and acoustic step-by-step guidance for hypoglycemic emergencies, as is used with modern defibrillators. Conventional training also has the potential to reduce errors in the administration of emergency medication [32].

Hypoglycemic emergencies can occur at any time, regardless of whether pediatric patients are supervised by their parents. Therefore, trainings also need to be provided for other caregivers, such as teachers and grandparents. Providing teachers with specific medical training not only reduces their fears of emergencies, but also increases the patients’ participation in school activities, such as excursions [13].

### 4.5. Limitations and Strengths

Some limitations have to be considered when interpreting the results. This study was conducted at one pediatric diabetes department; therefore, extrapolation to all of Germany or to other countries should be made with care. As participation was voluntary, parents with higher education and high confidence regarding their competence and skills might be overrepresented. Practical skills in glucagon administration were assessed using a demonstration model. We could not evaluate drug administration to patients during actual events of severe hypoglycemia.

One of the strengths of this study is its setting in real-life conditions at the families’ homes. Through this, we gained insight in actual storing conditions of glucagon. Another strength is the study protocol consisting of two methods: an interview combined with a practical demonstration. Furthermore, we were able to evaluate a drug administration that is difficult to access in clinical routine due to its nature as a rather rare emergency.

## 5. Conclusions

In our study, parents’ high and subjectively self-perceived competence in severe hypoglycemia management is contradicted by our findings by the objective expert assessment of their competence. Parents subjectively self-rated their skills in i.m./s.c. glucagon administration as acceptable. Their highly poor performance in the objective expert assessment of their skills shows a great need to improve drug safety.

The discrepancies between self-perception and objective expert assessment indicate a lack of error awareness that hinders improvement in hypoglycemia management and administration skills. To remove this barrier to optimal hypoglycemia management, practice-oriented training needs to be offered more frequently and targeted to individual deficits.

## Figures and Tables

**Table 1:** Characteristics of patients and parents.

	Patients (*n* = 140)	Parents (*n* = 140)
Age [years]: median (Q25/Q75; min/max)	11.0 (8.8/14.0; 3.0/17.0)	40.0 (37.0/44.3; 23.0/69.0)
Gender: *n* (%)		
Male	76 (54.3%)	29 (20.7%)
Female	64 (45.7%)	111 (79.3%)
Education: *n* (%)		
Not yet in school	16 (11.4%)	-
Primary school	42 (30.0%)	-
Middle school	37 (26.4%)	77 (55.0%)
Grammar school	38 (27.1%)	61 (43.6%)
Special needs school	5 (3.6%)	-
Vocational preparation year	1 (0.7%)	-
Vocational training	1 (0.7%)	-
Other	0 (0.0%)	2 (1.4%)
Professional education: *n* (%)		
University/university of applied sciences degree	-	48 (34.3%)
Completed vocational training	-	88 (62.9%)
Other	-	4 (2.9%)
Working in healthcare: *n* (%)		
Male	0 (0.0%)	0 (0.0%)
Female	0 (0.0%)	20 (14.3%)
Insulin therapy: *n* (%)		
Insulin pen	69 (49.3%)	-
Insulin pump	71 (50.7%)	-
Duration of diabetes [years]: median (Q25/Q75; min/max)	3.5 (1.4/6.5; 0.1/14.8)	-
Time since last training on hypoglycemia [years]: median (Q25/Q75; min/max)	-	2.3 (0.9/4.3; 0.1/11.8)
Previous use of glucagon in real-life emergency: *n* (%)	-	4 (2.9%)

**Table 2:** Drug handling errors in glucagon administration.

	Median Expert Rating of Clinical Risk (1: Low, 2: Moderate, 3: High)	Frequency *n* (%)
Administered dosage insufficient	3	54 (38.6%)
No adequate mixing of glucagon and solvent	3	24 (17.1%)
No injection of solvent into the vial	3	21 (15.0%)
Administered overdose (children < 25 kg)	3	9 (6.4%)
Intravenous administration	3	1 (0.7%)
Drawn liquid less than required amount of solution	3	57 (40.7%)
No verification if glucagon is dissolved	2	100 (71.4%)
Foaming of glucagon while mixing	2	65 (46.4%)
Needle removed from the vial while mixing	2	30 (21.4%)
Injection of less than required amount of solvent into the vial	2	24 (17.1%)
Hard plastic lid of the vial not removed before inserting the needle into the vial	2	21 (15.0%)
Air in syringe not removed	2	55 (39.3%)
Removal of the needle cap before removing the hard plastic lid of the vial	1	66 (47.1%)
Depositing the syringe with the needle cap removed	1	14 (10.0%)
Bare needle touched	1	13 (9.3%)

## Data Availability

The data presented in this study are available on reasonable request from the corresponding author. The data are not publicly available to protect the privacy of the study participants.
